# Application of Smartphone Otoscope in Telemedicine in Rural Medical Consortium in Eastern China in the COVID-19 Era

**DOI:** 10.3389/fpubh.2022.879410

**Published:** 2022-05-18

**Authors:** Xiangming Meng, Zhiyong Dai, Ying Wang, Xiang Hua, Xiaobo Gu, Jianxun Guo, Yangyang Wang, Chao Hang, Yuting Jiang

**Affiliations:** ^1^Department of Otolaryngology, Wuxi Huishan District People's Hospital, Wuxi, China; ^2^Wuxi Huishan District Qianqiao Street Community Health Service Center, Wuxi, China; ^3^Wuxi Huishan District Luoshe Town Shitangwan Health Center, Wuxi, China

**Keywords:** telemedicine, smartphone, otoscope, primary health care, medical consortium, WeChat, COVID-19

## Abstract

**Purpose:**

This study aimed to evaluate the effectiveness of smartphone otoscope telemedicine in the rural medical consortium in East China in the COVID-19 era.

**Methods:**

This prospective study was conducted within a rural medical consortium that provides health care services by integrating medical resources in the same area. When a patient visited primary health care (PHC) for ear diseases, the PHC provider used a smartphone otoscope to examine the patient's external ear canal and eardrum, and then sent photos or videos of the patient's ear to the otolaryngologist at the lead hospital *via* WeChat group. The otolaryngologist provided remote diagnosis and management recommendations to the PHC provider. The following data were recorded: age and gender, outpatient diagnosis, disease duration, sides, duration of treatment, telemedicine visits, treatment outcomes, patient satisfaction, and PHC providers' self-evaluation score.

**Results:**

A total of 83 patients were included in the study, including 43 males and 40 females, with a mean age of 44.6 ± 19.7 years (range 3–83 years). The duration of treatment for these patients was 14.0 (7,14) days. PHC visits were 2.2 ± 1.1 times (range: 1–7 times). Telemedicine visits ranged from 1 to 5, with a mean of 1.8 ± 0.9. Among of patients, 62 (74.7%) were cured, 21 (25.3%) improved, and 0 (0%) were ineffective. Sixty-five patients (78.3%) were very satisfied, 16 (19.3%) patients were somewhat satisfied, and two patients (2.4%) were dissatisfied. Based on the self-reported helpfulness, the primary health care providers assessed telemedicine as very helpful (*n* = 63, 75.9%), helpful (*n* = 20, 24.1%), and unhelpful (*n* = 0, 0%).

**Conclusions:**

Smartphone otoscope telemedicine in the medical consortium can effectively improve the ability of rural PHC providers to diagnose and treat ear diseases, save time and costs for patients, and improve patient satisfaction.

## Introduction

Primary health care (PHC) serves as the first point of contact for individuals and families in their community with a national health system by providing universally accessible, necessary health care services (1). China's PHC system guarantees that the general population access to comprehensive clinical care and essential public health services (1). However, physicians delivering PHC in community health centers frequently lack adequate training skills in some areas of medicine or some specialties. As a result, the efficacy of PHC has been compromised.

A medical consortium (MC) is a medical alliance created by integrating medical resources from the same region to deliver health care services (2, 3). The MC policy in China aims to improve the efficacy of health care delivery by encouraging collaboration among local health care professionals (4). Residents have accepted and recognized the approach (4). The rural MC is critical for improving the capability of primary medical services in rural areas (5). However, after the coronavirus disease 2019 (COVID-19) outbreak, rural PHC providers focused their efforts on tracing, screening, and educating critical public health responsibilities, which significantly influenced rural PHC operations (6).

Telemedicine, a component of digital health, is a rapidly growing area that employs communication technology to provide precise medical services to patients (7). Telemedicine and telehealth services have increased significantly in recent years, especially during the COVID-19 pandemic (8, 9). Smartphone apps have been applied to all aspects of health care, including telemedicine, cardiovascular disease prevention, older adults care, hearing screening, etc. (10–13). The smartphone otoscope (SO) is a relatively new electronic accessory device capable of capturing pictures and videos of patients' external auditory canal and tympanic membrane (TM) using a dedicated smartphone app (14). Image data can be stored on smartphones, computers, or shared over the Internet, allowing doctors, patients, and researchers to work closely. SO has been successfully applied in clinical diagnostics, telemedicine, procedural skills development, medical student education, and animal experiments (15–19). Our previous studies have shown that telemedicine with SO reduces outpatient visits, reduces the risk of cross-infection, and improves telemedicine accuracy during COVID-19 outbreak and the prevention and control phase of COVID-19 normality (14, 20). The telemedicine service was based on patients' self-examination with SO; unfortunately, some elderly patients were less able to use their smartphones, posing a challenge for telemedicine implementation (14).

Therefore, we endeavored to provide telemedicine services under the rural MC model by utilizing SO based on the WeChat platform. The purpose of the present study was to evaluate the effectiveness of smartphone otoscope telemedicine in the medical consortium (SOTITMC) in East China in the COVID-19 era.

## Patients and Methods

### Patients

This prospective observational study was conducted in a rural MC. The leading hospital of this medical association is Wuxi Huishan District People's Hospital, located in the western suburbs of Wuxi. It is a tertiary referral center and teaching hospital. Qianqiao Street Community Health Center (QSCHC) and Shitangwan Health Center (SHC), two PHC institutions in the same area, are members of this MC, which provides PHC services. Patients with ear disease who visited the outpatient departments of QSCHC and SHC from May 2021 to December 2021 were chosen. Patients who actively engaged in this telemedicine program were chosen, rather than all consecutive patients treated at PHC institutions. The inclusion criteria for patients were: (1) external ear or middle ear disease; (2) residents of the same community; (3) adults; and (4) children accompanied by their parents. Exclusion criteria were: (1) Patients who were residents from other communities or who came from outside the area for consultation; (2) Patients who were not interested in telemedicine; and (3) Patients who could not cooperate in completing the assessment of treatment effects.

According to the Declaration of Helsinki, the study was performed and granted by the Medical Ethics Committee of Wuxi Huishan District People's Hospital (Grant number: HYLL20210604001). All included individuals provided written informed consent. The informed consent form for children under 18 years of age was signed by the participant and by either a parent or a legal guardian.

### Telemedicine in Medical Consortium

The initial face-to-face diagnosis of the patients was performed in the outpatient settings of QSCHC or SHC. When PHC providers come across a patient who fits the eligibility requirements, they recommend enrollment in the MC telemedicine program. The Mebird T5 SO [Black Bee Intelligent Manufacturing (Shenzhen) Technology Co., Ltd., China] is an external ear canal or TM examination device. The device connects wirelessly to cellphones *via* a dedicated app. It features a built-in 3-megapixel camera that can take photos with a resolution of 2,048 × 1,536 pixels and videos with a quality of 480 × 480 P. Furthermore, the device comes with a directional gyroscope that maintains a steady angle of vision while the body rotates, making it simple to take correct ear photographs.

**Figure 1:**
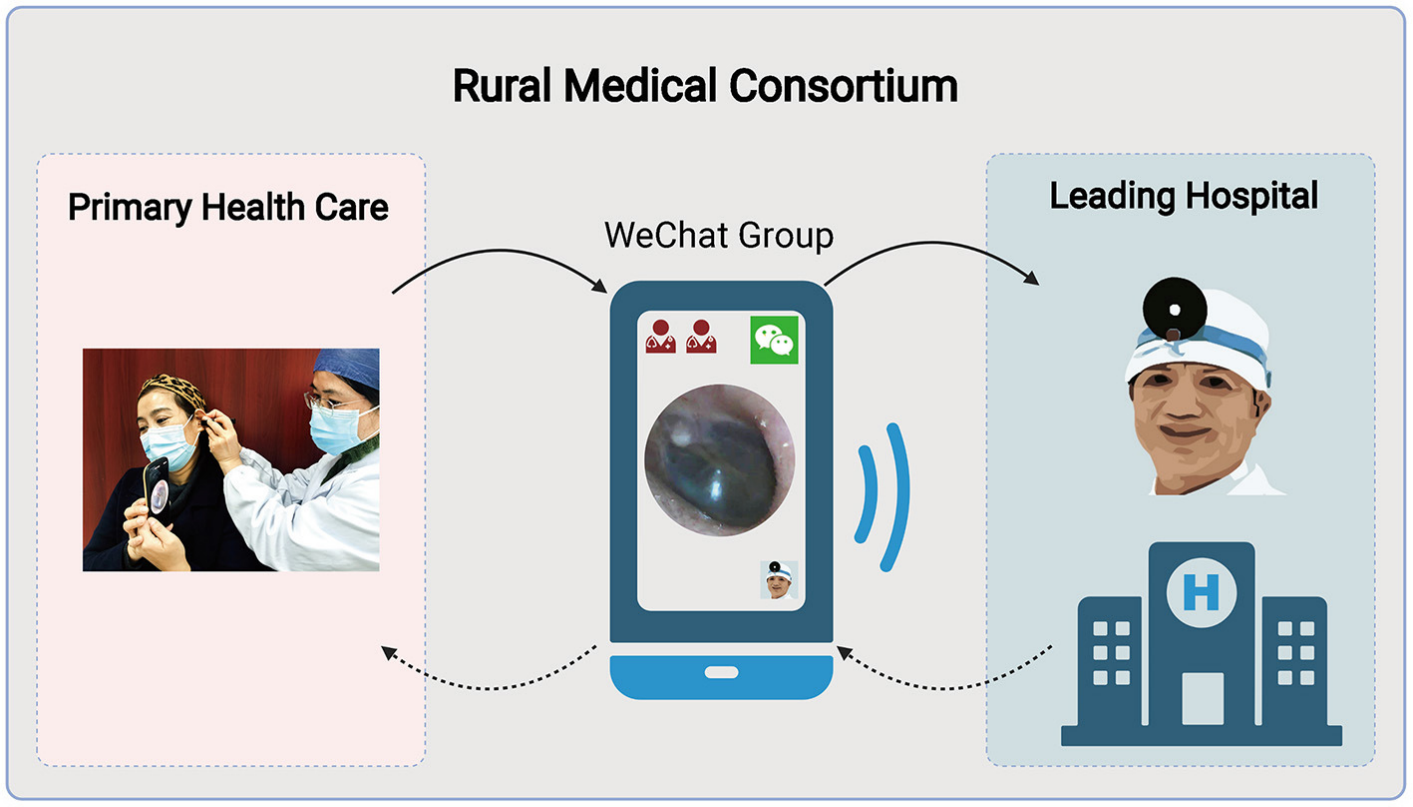
Flowchart of WeChat-based smartphone otoscope telemedicine within a rural medical consortium. The flowchart was created with BioRender.com.

During an initial visit, PHC providers collected information from patients, such as the primary complaint and complete medical history, inspected their ears with a SO, and recorded pictures and videos of their ears. Then, the PHC providers transmitted the photos and videos of their patients stored on their smartphones to the Medical Consortium Telemedicine WeChat Group (MCTWG). Meanwhile, information regarding the patient's condition was given to the MCTWG by text or voice. We have established the MCTWG exclusively for medical services in medical consortia. Otolaryngologists from leading hospitals and PHC providers are members of this group. Following the information alert in the MCTWG, the otolaryngologist analyzed the patient's pertinent information as soon as possible, offered a quick remote diagnosis, and provided treatment recommendations to the patient's PHC provider. Finally, PHC providers prescribed medications based on the advice of the otolaryngologists and scheduled patients for outpatient follow-up visits (Figure 1). This approach can complete the telemedicine visits in approximately 10 min. When the patient returned, they were still receiving telemedicine services in this manner. Following completing a patient's telemedicine, PHC providers and the otolaryngologist would remove the WeChat data held on their smartphones concerning the patient.

After completing one or more diagnoses and treatments for the disease, the PHC providers asked the patient to rate the face-to-face visit in combination with the telemedicine service based on their overall experience. Satisfaction was assessed using a simple visual analog scale ranging from 0 to 10, with 0 being the lowest level of satisfaction and 10 representing the highest level of satisfaction. Satisfaction scores ≤ 5 were rated “dissatisfied,” those with scores of 6–8 were rated “somewhat satisfied,” and those with scores ≥9 as “very satisfied.”

Primary health care providers rated themselves according to how they benefited from telemedicine for the disease in question. The helpfulness scores were divided into three categories: unhelpful (score ≤ 5), helpful (score 6–8), and very helpful (score ≥9).

Data recorded for all patients included patient age and gender, outpatient diagnosis, disease duration, sides, duration of treatment, telemedicine visits, treatment outcomes, patient satisfaction, and PHC providers' self-evaluation score. According to the clinical criteria, treatment outcomes were categorized as cured, improved, or ineffective.

### Statistical Analyses

All statistical analyses were performed with the R statistical software (Version 4.1.2, http://www.R-project.org). Continuous data were summarized using descriptive statistics; categorical data were summarized using counts and percentages. Age, telemedicine visits, and PHC visits were presented as mean ± standard deviation. Disease duration and duration of treatment were expressed as medians (25th percentile; 75th percentile).

## Results

### Patients

A total of 83 patients were enrolled in the study, 62 (*n* = 74.7%) of whom were from QSCHC and 21 (*n* = 25.3%) from SHC. Of these patients, 43 (*n* = 51.8%) were males, and 40 (*n* = 48.2%) were females, aged 3–83 years, with a mean of (44.6 ± 19.7) years. There were 38 cases (45.8%) on the left side, 33 cases (39.8%) on the right side, and 12 cases (14.5%) on both sides. The duration of ear disease was 6.5 (2,15) days. Characteristics of the patients are presented in Table 1.

**Table 1:** The characteristics of the patients and the outcomes of the treatment (*n* = 83).

**Characteristic and outcomes**	**Value**
PHC institutions [*n* (%)]	
QSCHC	62 (74.7%)
SHC	21 (25.3%)
Age (mean ± SD; years)	44.61 ± 9.7
Gender [*n* (%)]	
Male	43 (51.8%)
Female	40 (48.2%)
Sides [*n* (%)]	
Left	38 (45.8%)
Right	33 (39.8%)
Both	12 (14.5)
Disease duration (IRQ; days)	6.5 (2, 15)
Duration of treatment (IRQ; days)	14.0 (7, 14)
Telemedicine visits (mean ± SD; times)	1.80 ± 0.9
PHC visits (mean ± SD; times)	2.21 ± 0.1
Treatment outcomes [*n* (%)]	
Cured	62 (74.7%)
Improved	21 (25.3%)
Ineffective	0 (0%)

*IRQ, interquartile range; PHC, Primary health care; QSCHC, Qianqiao Street Community Health Center; SHC, Shitangwan Health Center*.

**Figure 2:**
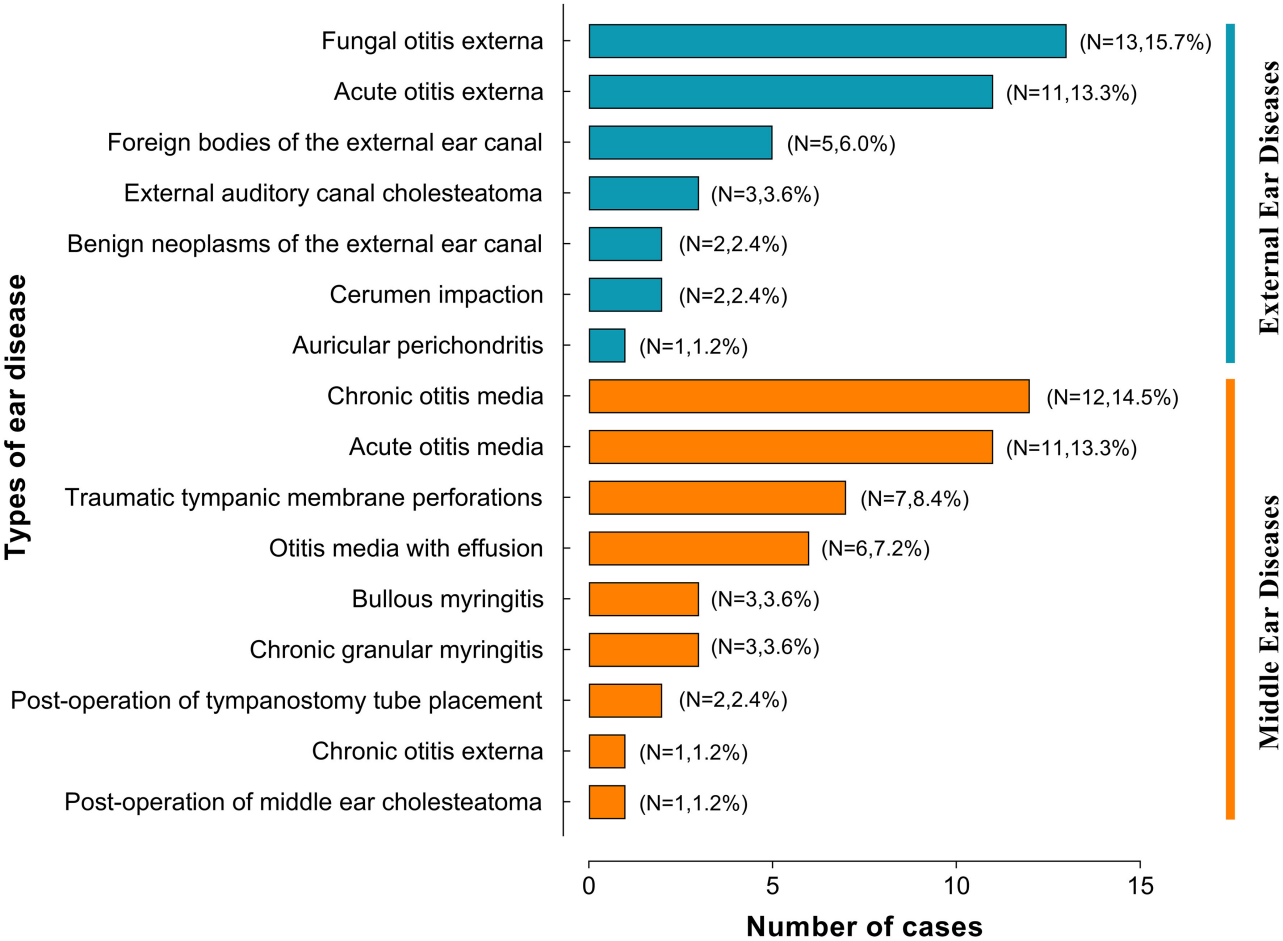
Types of ear disease in patients enrolled in a smartphone otoscope telemedicine study within a rural medical consortium.

The types of ear disease in the 83 participants who completed the study are shown in Figure 2. As we can see in Figure 2, the most involved disease types in this study were fungal otitis externa (FOE), chronic otitis media, acute otitis externa, and acute otitis media, in that order.

The mean duration of treatment for these patients in PHC was 14.0 (7,14) days. PHC visits was 2.2 ± 1.1 times (range: 1–7 times). Telemedicine visits by otolaryngologists ranged from 1 to 5, with a mean of 1.8 ± 0.9. The results showed that of 83 patients, 62 (74.7%) were cured, 21 (25.3%) improved, and 0 (0%) were ineffective. The treatment and outcomes of all patients are summarized in Table 1.

All but two patients were satisfied with the telemedicine service. Sixty-five patients (78.3%) were very satisfied, 16 (19.3%) patients were somewhat satisfied, and two patients (2.4%) were dissatisfied.

### Primary Health Care Providers

Primary health care providers stated they benefited from and improved their abilities due to telemedicine. Based on the self-reported helpfulness, the PHC providers assessed telemedicine as very helpful (*n* = 63, 75.9%), helpful (*n* = 20, 24.1%), and unhelpful (*n* = 0, 0%).

## Discussion

In the present study, we investigated the effectiveness of SOTITMC. To the best of our knowledge, it is the first study involving SO in a rural MC for telemedicine applications. Most patients have expressed satisfaction with this telemedicine service, which is likely due to improved treatment outcomes and convenience of access to health care. SOTITMC improved diagnosis and treatment in PHC while also facilitating skills training for PHC providers, according to our findings. The PHC providers were satisfied with what they learned from all the telemedicine encounters when dealing with their patients.

Although the number of patients with otitis media decreased during the epidemic compared to the previous period, otitis externa and otitis media were still common in PHC. FOE was ranked first in this study, which might be related to the MC's geographical location and climatic environment. Wuxi city is the subtropical monsoon climate zone, with sufficient heat and abundant precipitation (21). This climatic characteristic causes an increased incidence of FOE. FOE is more common in hot, humid climates (22).

Previous studies have shown that SO plays a beneficial role in clinical diagnosis and telemedicine. Mousseau et al. (15) found that the accuracy of SO in diagnosing acute otitis media in young children was comparable to that of using conventional otoscopy in the pediatric emergency department of a tertiary care hospital. Don et al. (16) revealed that SO for tympanostomy tube monitoring is workable, allowing otolaryngologists to remotely track children's tympanostomy tubes and providing greater parental satisfaction. With high severe acute respiratory syndrome coronavirus 2 (SARS-CoV-2) viral load in the nose and throat and anosmia as one feature of COVID-19, otolaryngology is a high-risk department for COVID-19 (9, 23, 24). As a result, several studies used SO for telemedicine services during COVID-19. Bayoumy et al. reported a case of self-monitoring in a patient with surgically corrected TM retraction, suggesting that SO can be a worthy addition to regular follow-up (9). A digital USB otoscope (model D13L22) was used to obtain good images of the TM in this case report (9). A previous study we conducted also investigated patient satisfaction with SO telemedicine, with 71.9% of patients being very satisfied and 28.1% being somewhat satisfied (14). The SO model used in our previous study was the Mebird M9pro, manufactured by the same company as the SO used in the present study, which has similar imaging quality (14).

This study has brought many benefits to the clinical practice of PHC. First, SOTITMC has expanded and strengthened its technical assistance to medical institutions members of the MC. Before the COVID-19 outbreak, an otolaryngologist from the lead hospital scheduled weekly visits to QSCHC and SHC, both PHC institutions, to provide routine outpatient services and technical support. However, due to the COVID-19 pandemic, this technical collaboration became erratic and even discontinued for an extended period, negatively impacting PHC. This strategy compensated for the absence of face-to-face expert visits. Second, SO made up for the lack of equipment for otolaryngology examinations in rural PHC institutions. The ear has complex anatomy that necessitates specialized equipment to inspect.

With SO, the PHC providers can accurately examine a patient's external ear canal and TM and send the information to an otolaryngology specialist. The otolaryngology specialist could visualize the patient's ear performance with still photos and accurately judge dynamic performance, such as the mobility of the TM, with video data. Third, WeChat is the most popular communication application in China, with over 1 billion users (25). WeChat employs the Client-Server encryption security model to ensure security, encrypting the entire database SQLite file with a 256-bit long key and the AES encryption technique (26). The 4G mobile phone network in China has become popular, and some users are already using 5G phones. The robust mobile phone network ensured that image data was delivered quickly. The otolaryngology specialist and PHC providers could communicate and exchange ideas in a timely and effective manner through the MCTWG. In other words, SOTITMC allows otolaryngologists to provide access to PHC providers with around-the-clock technical guidance *via* the Internet. Fourth, SOTITMC saved the time and cost for the patients and provided them with convenient access to medical services. Since the COVID-19 outbreak, the health authorities in China have been implementing strict preventive and control measures (27). Even under the control of COVID-19, the hospitals also implemented standard measures, such as reviewing health QR codes and healthy travel routes, monitoring body temperature, etc. (14). It took more time for patients to visit the hospitals. Patients attended a community PHC institution closer to their home rather than being referred to a tertiary hospital farther away from their home. It saved time and transportation costs and reduced the aggregation and mobility of the population, which facilitates the prevention and control of pandemics. Furthermore, the ethical and legal implications of storing patient data on smartphones should be considered (8). Since the WeChat connection is not end-to-end encrypted, the use of WeChat may not comply with the relevant laws and regulations in some countries, so it is essential to consider this when practicing telemedicine.

Despite its many strengths, the present study has some limitations. There was a relatively large gap between the image quality of a SO and that of an otoscope equipped with a high-definition camera. It might affect the ability of the otolaryngologist to identify minor lesions in the ear of the patient. In addition, the number of patients included in this study was not large enough. Because of the frequent assignment of PHC providers who participated in this work to COVID-19 pre-screening triage, COVID-19 vaccination, and COVID-19 isolation sites, the SOTITMC program was frequently interrupted, resulting in a limited sample size. The included patients were not successive, and there might have been selection bias. We need to gain more experience with SO telemedicine by having more patients in the future. Additionally, the otolaryngologists at the leading hospital documented fewer patient data in this study, which has to be improved.

## Conclusion

In summary, SOTITMC can effectively improve the ability of PHC providers in the rural MC to diagnose and treat middle and external ear diseases, save time and costs for patients, and improve patient satisfaction. It has also facilitated skills training for PHC providers, with positive feedback. In addition, this telemedicine modality reduces population aggregation and mobility, decreases the risk of cross-infection, and provides new ideas for preventing and controlling the ongoing COVID-19 pandemic.

## Data Availability Statement

The original contributions presented in the study are included in the article/supplementary material, further inquiries can be directed to the corresponding author/s.

## Ethics Statement

The studies involving human participants were reviewed and approved by Medical Ethics Committee of Wuxi Huishan District People's Hospital. Written informed consent to participate in this study was provided by the participants' legal guardian/next of kin.

## Author Contributions

XM: conceptualization, methodology, data curation, project administration, funding acquisition, and writing—reviewing and editing. ZD: methodology and project administration. YiW, XH, XG, JG, and YJ: investigation and data curation. YaW: investigation and software. CH: supervision and formal analysis. All authors contributed to the article and approved the submitted version.

## Funding

This work was supported by the Soft Science Research Project of Science and Technology Association of Wuxi Municipality (Grant number: KX-21-B64) and the Appropriate Technology Promotion Project of Wuxi Municipal Health Commission (Grant number: T202119), China.

## Conflict of Interest

The authors declare that the research was conducted in the absence of any commercial or financial relationships that could be construed as a potential conflict of interest.

## Publisher's Note

All claims expressed in this article are solely those of the authors and do not necessarily represent those of their affiliated organizations, or those of the publisher, the editors and the reviewers. Any product that may be evaluated in this article, or claim that may be made by its manufacturer, is not guaranteed or endorsed by the publisher.

## Abbreviations

PHC: primary health care
MC: medical consortium
COVID-19: coronavirus disease 2019
SO: smartphone otoscope
TM: tympanic membrane
SOTITMC: smartphone otoscope telemedicine in the medical consortium
QSCHC: Qianqiao Street Community Health Center
SHC: Shitangwan Health Center
MCTWG: Medical Consortium Telemedicine WeChat Group
Fungal otitis externa (FOE)
SARS-CoV-2: Severe acute respiratory syndrome coronavirus 2.
